# Fat storage in *Drosophila suzukii* is influenced by different dietary sugars in relation to their palatability

**DOI:** 10.1371/journal.pone.0183173

**Published:** 2017-08-17

**Authors:** Maurizio Biolchini, Elisabetta Murru, Gianfranco Anfora, Francesco Loy, Sebastiano Banni, Roberto Crnjar, Giorgia Sollai

**Affiliations:** 1 Department of Biomedical Sciences, Section of Physiology, University of Cagliari, Monserrato (CA), Sardinia, Italy; 2 Department of Biomedical Sciences, Section of Cytomorphology, University of Cagliari, Monserrato (CA), Sardinia, Italy; 3 Center Agriculture Food Environment, University of Trento, Trento, Italy; Inha University, REPUBLIC OF KOREA

## Abstract

The peripheral sensitivity and palatability of different carbohydrates was evaluated and their nutritional value assessed in adult females of *D*. *suzukii* by means of an electrophysiological, behavioural and metabolic approach. The electrophysiological responses were recorded from the labellar “l” type sensilla stimulated with metabolizable mono- and disaccharides (glucose and maltose) and a non-metabolizable sugar (sucralose); the response rating and the palatability to the same sugars, evaluated by recording the proboscis extension reflex (PER), was maltose>glucose>sucralose. The nutritional value of carbohydrates was assessed by means of survival trials and fatty acids profile. Flies fed on a diet containing maltose had a longer lifespan than flies on monosaccharides, while flies fed on a diet containing sucralose had a shorter one. In addition, the ability to store fat seems to be influenced by the different sugars in the diet and is in relationship with their palatability. In fact, data showed a higher synthesis of palmitic and palmitoleic acids, most likely derived from *de-novo* lipogenesis with glucose as precursor, in flies fed with maltose and glucose than with non-metabolizable sucralose. In conclusion, these results suggest that the ability to select different sugars on the basis of their palatability may favour the storage of energy reserves such as fat by *de-novo* lipogenesis, determining a longer survival capability during prolonged periods of fasting.

## Introduction

In insects, the behaviour of host selection is strongly influenced from sensory input coming from their chemical senses [1–4]. In particular, the taste sensory system plays a central role in identifying and evaluating potential foods by discriminating between nutritious chemicals that promote feeding, and structurally diverse, harmful, or even toxic compounds, that inhibit feeding [5,6].

Insects offer several advantages for the study of peripheral taste sensitivity. Unlike the case of vertebrates, taste transduction is performed by bipolar neurons called gustatory receptor neurons (GRNs), housed within bristles called taste sensilla, located on the labellum, legs, wing margins and ovipositor [7,8]. Taste sensilla have an apical pore that enables to record the neural activity originating from single GRNs [9]. The responses of these cells have been well characterized: the spikes recorded from each of the four chemoreceptors consistently differ in amplitude and shape. They can be separated from one another and their relative amplitude ratios are conserved; they are species- and sensillum-type specific regardless of recording conditions [1,10,11].

In *Drosophila melanogaster* labellar taste sensilla have been classified on the basis of their length and their distribution, into three main classes: small (“s” type), long (“l” type) and intermediate (“i" type [12,13]. The **“**s” type and “l” type sensilla contain four bipolar GRNs, whereas “i" type sensilla bear only two GRNs [14,15].

The GRNs fall in 4 functional classes: the S cell is activated by sugars (mono-, di- and trisaccharides), the W cell is tuned to water and L1 and L2 cells are activated by low salt and high salt, respectively [12,16–18]. The “i" type sensillum lacks the W cell and a single GRN has both L1 cell and S cell properties, while a second GRN has L2 cell properties [12]. The L2 cell in “i" type and “s” type sensilla is activated not only by high salt but also by bitter compounds and low pH solutions [19–21].

The stimulation of labellar or tarsal taste neurons with an attractive stimulus such as a sugar determines the proboscis extension, the spreading of the labella and the start of feeding. On the contrary, the addition of an unpleasant compound to the food source suppresses the proboscis extension reflex (PER) and elicits retraction.

An important feature of the neural circuits that control feeding is that they integrate information about palatability (taste) and nutritional content of the food source. These variables are often related to each other [22]; in fact, sugars generally represent a convenient source of carbohydrates and have a palatable sweet taste. However, the intensity of the sensory response to a specific sugar is not always indicative of its nutritional value, and recent data suggest that insects can detect the caloric content of food regardless of taste [5,23–25].

In *D*. *melanogaster*, energy substrate reserves in the form of glycogen and triglycerides are stored in fat body cells, an important tissue that plays a major role in the life of insects and is involved in multiple metabolic functions [26,27]. The lipid content is influenced by many factors such as stage of development, nutritional state, sex, environmental temperature, diapause and migratory flight [28]. The insects possess a period of rapid lipogenesis during which dietary carbohydrates may be converted to lipids and stored in the fat body as triglycerides [29–31]. *De novo* lipogenesis (DNL) in the fat body follows a similar pathway as in the mammalian tissues [32]. The efficiency of lipogenesis from carbohydrates is much higher than that for glycogen synthesis, which explains the higher content of lipids as compared to glycogen in the fat body [33].

The advantages of storing lipids over carbohydrates as metabolic fuel include a higher caloric content per unit weight of substrate and thus the use of lipids as a primary metabolic substrate permits accumulation of a large reservoir of energy which may be used during periods of prolonged energy demand [33].

Within the "melanogaster group", *Drosophila suzukii* Matsumura (Diptera: Drosophilidae) is a polyphagous insect, with a broad climate range tolerance and high invasive potential. Unlike *D*. *melanogaster* that lays eggs and feeds only on decaying and rotten fruits, *D*. *suzukii* lays eggs and feeds on unripe and undamaged fruits [34–36]. This difference in ecology is reflected in neurological and physiological adaptations to finding, and feeding on, unripe food sources [37].

On the basis of these considerations, the aim of this study was to evaluate peripheral sensitivity and palatability in relation to nutritional value of different carbohydrates, in adults of *D*. *suzukii* by means of an electrophysiological, behavioural and metabolic approach. First, the spikes activity was recorded from the labellar l-type sensilla following stimulation with two metabolizable mono- and disaccharides (glucose and maltose) and one non-metabolizable sugar (sucralose); second, the palatability of the same sugars was evaluated by recording the PER activity; finally, the nutritional value of the carbohydrates was evaluated by means of survival trials, and lipid content, while fatty acids profile was used to assess the ability to store fats by DNL.

## Materials & methods

### Insects

Four to ten-day adults of *Drosophila suzukii* (Diptera: Drosophilidae) were obtained from lab-reared colony at the Dept. of Biomedical Sciences of the University of Cagliari (Italy). In the larval stage, flies were fed on Drosophila standard diet [38] under controlled conditions (23°C, 70% of relative humidity, 14L/10D photoperiodic regime).

### Morphological observations

Adults of *D*. *suzukii* were observed by a Hitachi S4000 Field Emission Scanning Electron Microscope operated at 20 kV. In order to fix the tissues and avoid collapse of the mouthparts, insects were injected with a mixture of 1% paraformaldehyde and 1.25 glutaraldehyde in 0.15 M cacodylate buffer at room temperature for 30 min. After washing in bidistilled water, samples were sonicated twice in a Triton X-100 1% solution and then dehydrated in acetone. Samples were later critical point dried and coated with 2 nm platinum by means of an Emitech K575 Sputter Coater [39]. Photos were collected by a Quartz PCI v. 5 software (Quartz Imaging Corporation, Vancouver, BC, Canada).”

### Stimuli

Taste solutions were prepared immediately before testing and were presented at room temperature (23°C). The following sugars were tested: maltose, glucose and sucralose. They were added with tricholine citrate to provide adequate conductivity to the stimulating/recording solution. All compounds were purchased from Sigma-Aldrich, (Italy).

### Electrophysiological experiments

Electrophysiological recordings were performed from the apical pore of the “l” type labellar sensilla, by means of the “tip-recording” technique [9]. Recording operations were carried out by means of micromanipulators under the field of a stereomicroscope. The reference electrode, a thin Ag/AgCl, was inserted into the base of the isolated head to fix the labellum in prognathous position. The recording electrode, a glass micropipette (tip diameter 20 μm), containing the stimulating solution, was brought in contact with the sensillum tip. All signals were recorded with a high input impedance (10^15^ Ω) electrometer (WPI, Duo 773), band-pass filtered (0.1–3 KHz), digitized by means of an Axon Digidata 1440A A/D acquisition system (sampling rate 10 KHz) and stored on PC for later analysis [40].

Stimuli were applied in a randomized sequence and a 3-min interval was allowed between consecutive stimulations to minimize adaptation phenomena. Each sensillum was tested with aqueous solutions of 1÷100 mM maltose, glucose and sucralose in the dose-response experiments, and with 100 mM of each sugar in the taste sensitivity ones. All taste stimuli were dissolved in 30 mM tricholine citrate (TCC), which was also tested alone as control [18,21]. The 30 mM TCC (control solution) was tested at the beginning and the end of each recording sequence to check for shifts in responsiveness. In order to avoid any drift in solution concentration due to evaporation, a clean, dry piece of filter paper was used to draw fluid from the tip of the recording/stimulating electrode just before each recording. After each test, the labellum was rinsed with distilled water and blotted dry.

### Data analysis

Recordings typically lasted 2–3 s, but spike analysis was performed in the interval 10–1010 ms after contact with the sensillum, the first 10 ms being skipped as containing the contact artefact. The first second of the discharges was chosen as representative of the phasic/phasic-tonic sections of the response [41–43]. The spike sorting and counting were performed by means of the Clampfit 10.0 software, based on earlier studies [44–47]. By measuring the peak-antipeak spike amplitude we identified three different spike types that were labelled as small (S), medium (M) and large (L), in response to sugar solutions and two spike types (S, M) in response to TCC alone, added as a conducting agent to all stimuli (S1 and S2 Figs).

### Behavioural experiments

#### Proboscis extension reflex (PER)

PER experiments were performed according to Dahanukar et al. [48] and Burke et al. [23]. Briefly, flies were food deprived for 24 hr inside vials in the presence of water. Flies were trapped into a p200 pipette with the tip cut to expose the head and forelegs to stimuli. Each tip was held upright on a slide by a piece of clay and was positioned under a stereomicroscope. After 5 min, each fly was observed through the objective of the microscope, and its PER responses were counted. A piece of filter paper was moistened with the sugar solution (100 mM) dissolved in bidistilled water and was brought in contact with the labellar sensilla for 2 s. The sequence of stimulations included a negative control (water), a sugar stimulus and a positive control (2M sucrose). Test stimuli were presented 3 times per fly and each fly was tested with all three stimuli (N = 33 flies/sugar). Flies which showed PER to water alone or that failed to extend to 2M sucrose at the end were discarded from the analysis. PER responses were scored as follow: full extension = 100, half or weak extension = 50, no extension = 0.

#### Capillary feeder assay (CAFE)

A modified capillary feeding assay (CAFE) was used to measure food intake in no-choice trials and feeding preference in two-choice trials, according to Masek et al. 2013 [49] and Diegelmann et al. 2017 [50]. Briefly, three to five-day adult flies were divided into 5 groups of 20 flies per assay and starved for 24 hr inside a plastic vial containing only a layer of agar solution (1.5%), as a water source. After starvation, flies were allowed to drink from two 5μL capillaries (Blaubrand), filled with the test solution, held in place by truncated p200 pipette tips inserted in two holes in the vial plastic lid. Both no-choice and two-choice trials were performed in a climatic chamber under controlled conditions (23°C; RH 70%), from 14:00 to 17:00 pm in the case of no-choice and from 14:00 to 16:00 pm, in total darkness, in the case of two choice trials. Water loss due to evaporation was measured in three empty vials, identical to those used in the assays, provided with two capillaries filled with water. In the case of no-choice trials, capillaries were filled with the sugar solution at a concentration equivalent to that used for survival trials (43.82 mM) mixed with a blue food dye (Food Blue No.1) to better visualize the amount of consumed solution.

In the case of two-choice trials, capillaries were filled with the sugar solution (100 mM) mixed with blue food dye (Food Blue No.1) or red dye (Food Red No.40) at a concentration of 3μL per 1 mL of solution [49]. A thin layer of mineral oil (Thermo Scientific) covering the top of the capillaries was used to prevent evaporation.

For both no-choice and two-choice trials, the amount of consumed liquid from each capillary was measured with a ruler and the value obtained converted to a volume of food eaten (15 mm of consumed solution in the capillary = 1 μL of solution).

Food consumption (μL) per each fly was calculated as food uptake (μL) minus evaporative loss (μL), divided by the total number of flies in the vial [50].

#### Survival trials

Immediately after eclosion, flies were separated in 3 groups and each of them was fed on the standard diet that varied only by the sugar type. Each diet contained a sugar amount equivalent to that of the sucrose present in the standard diet (43.82 mM). A fourth group of flies was fed on the standard diet without sugars (starved flies). For each diet, 50 flies per three repeats were allowed to feed ad libitum for 72h e then were divided into 5 vials containing only water (10 flies/vial). The number of dead insects was counted every 12h from the beginning of the trial, for a period of 48h.

### Metabolic experiments

Immediately after eclosion (day 0) 25 flies were sacrificed and their fatty acid profile, accumulated during the larval stage was evaluated (first control). 175 flies were divided into 7 groups (N = 25/group): 6 of them were fed *ad libitum* for 72h on the standard diet that varied only by the sugar type (maltose, sucrose, glucose, fructose, sucralose or arabinose), the last one was fed on standard diet without sugar (internal control to check whether the amount and composition of fatty acids was linked to other components of the diet besides sugar). Since we did not find any significant differences in the amount of total FAs after the flies had been fed on a diet containing one of the two disaccharides maltose/sucrose, or one of the two monosaccharides glucose/fructose, or one of the two non-metabolizable sugars sucralose/arabinose, we decided to choose only one for each sugar typology: maltose as disaccharide, glucose as monosaccharide and sucralose as non-metabolizable sugar, in order to evaluate any variation in fatty acid profiles. Consequently, four more groups (N = 10 flies/group) were fed *ad libitum* for 72h on a diet without sugar or with added maltose or glucose or sucralose, and thereafter, were food-deprived for 36h. Their fatty acid concentrations in alive flies were then analyzed.

Total lipids were extracted by the method of Folch et al. [51]. Aliquots of the lipid fraction were mildly saponified using a procedure in order to obtain free fatty acids for HPLC analysis [52]. Separation of unsaturated fatty acids was carried out with an Agilent 1100 HPLC system (Agilent, Palo Alto, CA, USA) equipped with a diode array detector. A C-18 Inertsil 5 ODS-2 Chrompack column (Chrompack International BV, Middleburg, The Netherlands), 5 μm particle size, 150 mm×4.6 mm, was used with a mobile phase of CH_3_CN/H_2_O/CH_3_COOH (70/30/0.12, v/v/v) at a flow rate of 1.5 ml/min [53]. Because saturated fatty acids (SAFAs) are transparent to UV, they were measured, after methylation [54], by means of a gas chromatograph (Agilent, Model 6890) equipped with split ratio of 20:1 injection port, a flame ionization detector, an autosampler (Agilent, Model 7673), a 100m HP-88 fused capillary column (Supelco) and an Agilent ChemStation software system. The injector and detector temperatures were set at 250°C and 280°C, respectively. H_2_ served as carrier gas (1 mL/min) and the flame ionization detector gases were H_2_ (30 mL/min), N_2_ (30 mL/min), and purified air (300 mL/min). The temperature program was as follows: initial temperature was 120°C, programmed at 10°C/min to 210°C and 5°C/min to 230°C, then programmed at 25°C/min to 250°C and held for 2 min.

### Statistical analysis

Repeated-measures ANOVA was employed to analyze the effect of increasing sugar concentration on the spike frequency of discharges recorded from GRNs in the labellar “l” type sensilla, for each stimulus. One-way ANOVA was used to analyze: a) the effect of sugar on spike frequency in the first second of discharges; b) the effect of sugar on the activity of the PER; c) the effect of sugars on the amount of food intake in double choice conditions; d) the effect of sugar on the amount of total fatty acids (FAs), SAFAs, monounsaturated fatty acids (MUFAs) and polyunsaturated fatty acids (PUFAs) and individual fatty acids. Two-way ANOVA was used to analyze the interaction of Feeding substrate x Time on flies survival.

Data were checked for the assumptions of sphericity, homogeneity of variance and normality. When the sphericity assumption was violated, a Green-Geisser correction or Huynh-Feldt correction was applied in order to modify the degrees of freedom. Post-hoc comparisons were conducted with the Tukey test, unless the assumption of homogeneity of variance was violated, in which case Duncan’s test was used. Statistical analyses were performed using STATISTICA for WINDOWS (version 7.0; StatSoft Inc, Tulsa, OK, USA). P values < 0.05 were considered significant.

## Results

### Map of labellar sensilla

**Fig 1:**
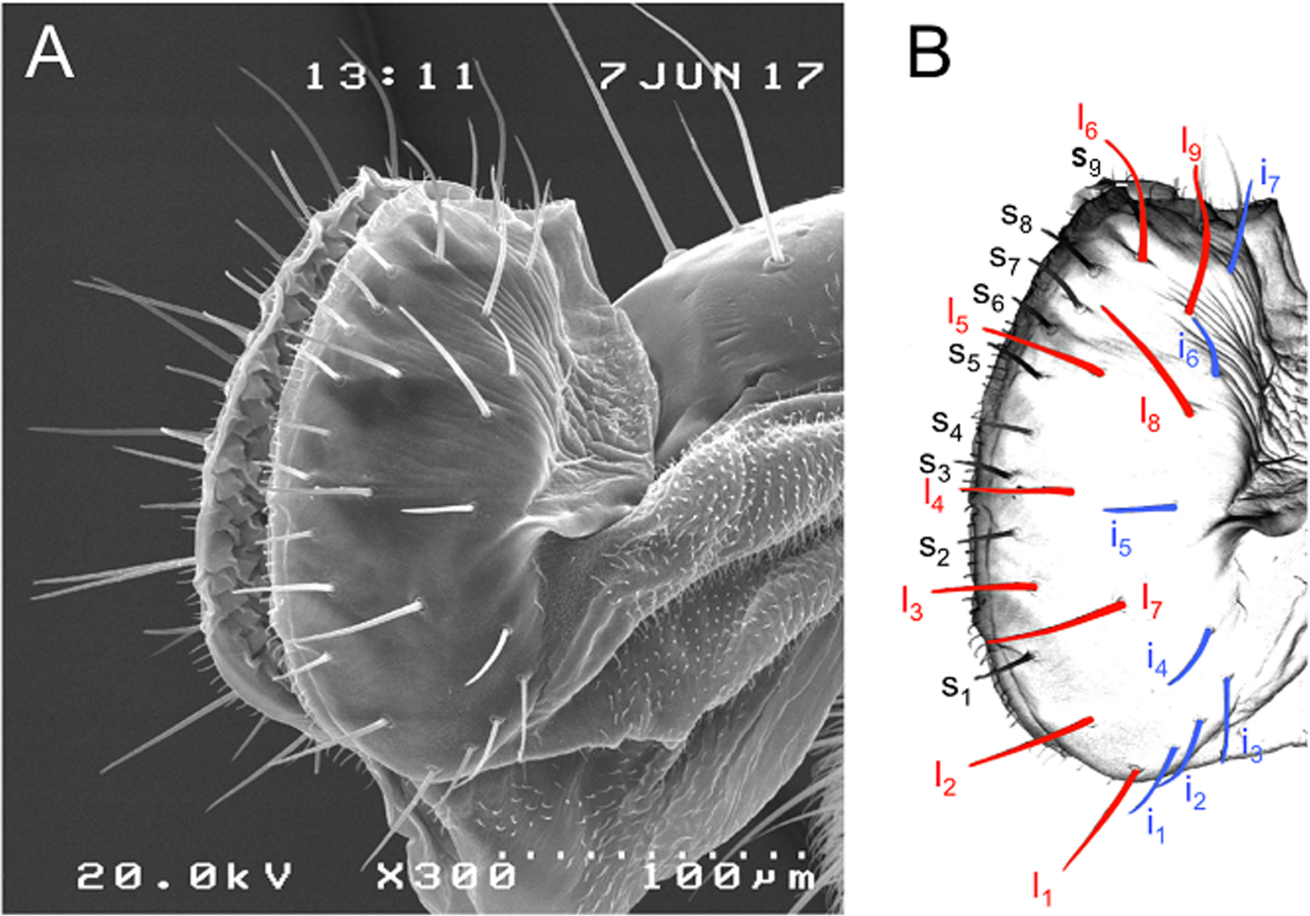
(A) SEM image of a proboscis hemilabellum of *Drosophila suzukii* showing the distribution pattern of taste sensilla. (B) Corresponding schematic arrangement of the three sensillum types: small (s_1-9_), intermediate (i_1-7_) and large (l_1-9_).

Fig 1 shows the map of labellar taste sensilla in *Drosophila suzukii*. As in *D*. *melanogaster* [20], there are three types of sensilla: small (s), intermediate (i) and large (l). Their distribution pattern is similar in the two species. Besides, in *D*. *suzukii* each hemilabellum presents a lower number of sensilla of the “s” and “i” types than in *D*. *melanogaster* (9 vs. 11 and 7 vs. 11 respectively), but the same number of “l” type sensilla (9).

### Dose-response profiles and taste sensitivity to sugars

**Fig 2:**
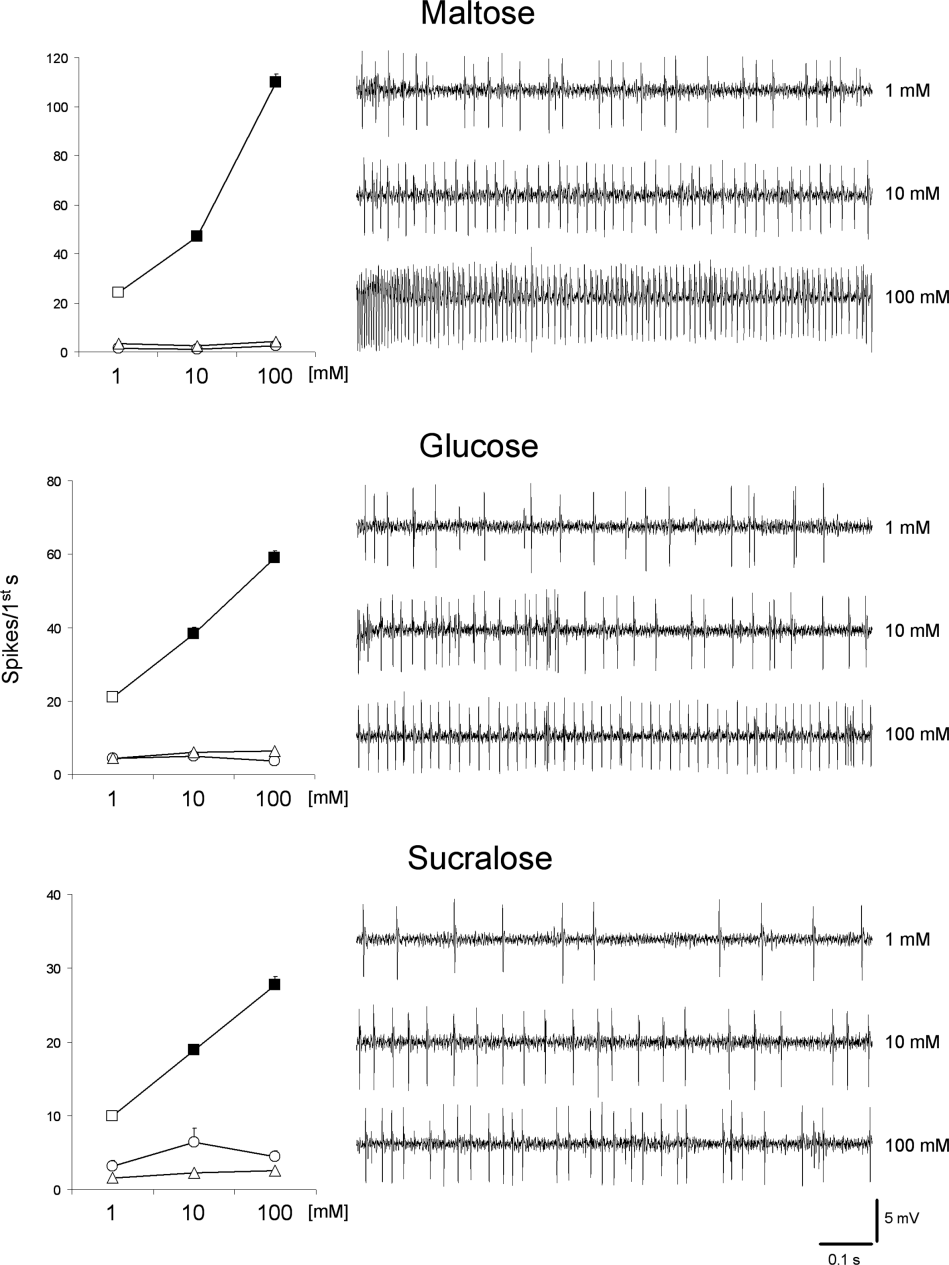
Characterization of sugar GRN in "l" type sensilla. Samples of spike discharges and dose-response relationship between spike activity of GRNs of an "l" type sensillum and 1÷100 mM maltose, glucose and sucralose. Mean values ± s.e.m. N = 32–34. Filled symbols indicate significant differences between a concentration and the next lower (P<0.001; Tukey test subsequent to repeated measures ANOVA). Different spike types are indicated by the following symbols: square = "L" spike; triangle = "M" spike; circle = "S" spike.

Samples of the GRN spike activity, recorded from the “l” type labellar sensilla, in response to increasing concentrations of maltose, glucose and sucralose are shown in Fig 2.

To test for a dose-response relationship, we analyzed the spike activity evoked in the first second of the discharge to increasing concentrations of each sugar, by using a repeated-measures ANOVA (Fig 2). Repeated- measures ANOVA revealed a significant effect of concentration on the spike frequency of the “L” neuron in response to maltose (F_[1.2,40]_ = 413.26; P<0.00001), glucose (F_[__2__,__62__]_ = 103.97; P<0.00001) and sucralose (F_[1.7,52.4]_ = 121.01; P<0.00001). Post-hoc comparisons showed that the spike activity in response to each concentration was lower than in response to the next higher concentration, for each sugar tested (P<0.001; Tukey test). No effect of the concentration was found for neurons “S” and “M” (maltose: F_[__2__,__66__]_<2.5608; P>0.05; glucose and sucralose: F_[__2__,__62__]_<1.6176; P>0.05). These results show that “L” neuron is activated by sugars.

**Fig 3:**
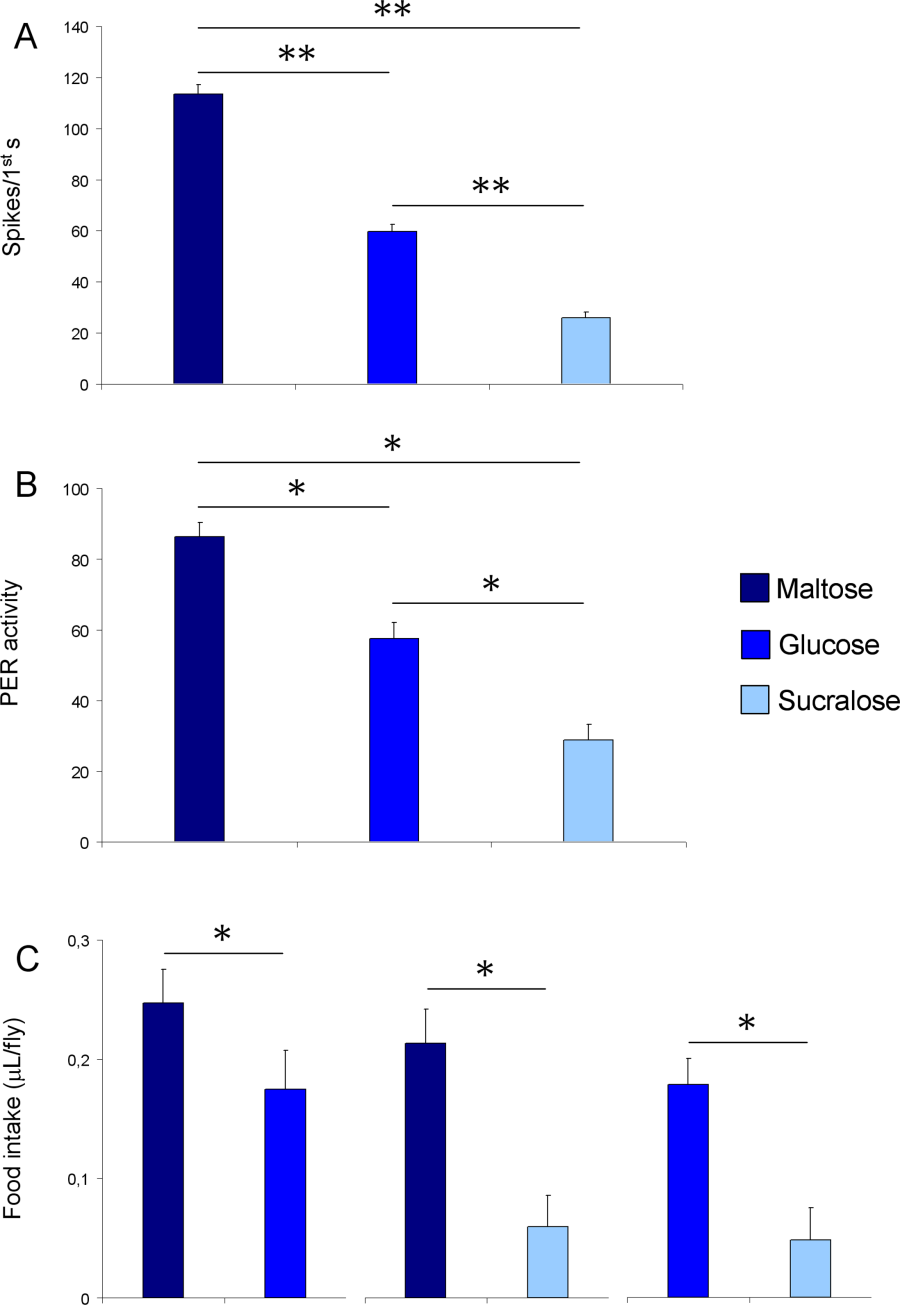
Electrophysiological, PER and CAFE results. A) Mean values ± s.e.m. of spike frequency of “sugar” GRN following stimulation with 100 mM maltose, glucose and sucralose. N = 45–50. B) Mean values ± s.e.m. of PER activity following stimulation of labellar sensilla with 100 mM maltose, glucose and sucralose. N = 33. C) Mean values ± s.e.m. of the amount of maltose, glucose or sucralose intake under double-choice conditions. N = 5. * = P<0.05; ** = P<0.005.

To test the differences in taste sensitivity to different sugars, we analyzed the spike activity of the “L” neuron in the first second of the discharges to 100 mM of each sugar, by using one-way ANOVA (Fig 3A).

One-way ANOVA showed a significant effect of the stimulus on the frequency of the L-type spike (F_[__2__,143]_ = 169.90; P<0.00001) and post-hoc comparisons showed that the L-type spike frequency in response to maltose was higher than in response to glucose and sucralose (P<0.0001 for both sugars; Duncan’s test). Besides, spike frequency in response to sucralose was lower than in response to the other two sugars (P<0.0001; Duncan’s test). These findings indicate that, in the “l” type labellar sensilla, the effectiveness of the sugars tested is: maltose > glucose > sucralose. At the beginning of the analysis, we cross-compared the spike responses of the different “l” type sensilla (l_1_ to l_9_), but we did not find any differences, so in the final analysis we did not take into account the sensillum position. For example, we tested the differences in the spike activity of l_2_, l_4_ and l_5_ sensilla following stimulation with 100 mM maltose, glucose and sucralose (S3 Fig). One-way ANOVA revealed no effect of the sensillum number on the spike frequency (F_[__2__,__17__]_<0.20499; P>0.05).

### Palatability of sugars

One-way ANOVA was used to test quantitative differences both in the activity of proboscis extension reflex following stimulation of the labellar sensilla with the various sugars tested (Fig 3B) and in the amount of food intake in a double-choice condition (Fig 3C): a significant effect of the stimulus on both PER activity (F_[__2__,__64__]_ = 43.429; P<0.00001) and amount of food intake was found (F_[__1__,__8__]_>5.5632; P<0.05). Post-hoc comparisons revealed that flies displayed high levels of PER in response to maltose as compared to other sugars (P<0.001; Tukey test); sucralose elicited a significantly lower PER than the other 2 sugars (P<0.001; Tukey test). Finally, glucose elicited a PER level intermediate between the other sugars. Besides, post-hoc comparisons also revealed that the amount of maltose eaten was significantly higher than both glucose and sucralose (P<0.05; Tukey test), while that of sucralose was significantly lower than the other two sugars (P<0.01; Tukey test). These findings indicate that the palatability of sugars tested is: maltose > glucose > sucralose.

### Effect of sugars on fly survival

**Fig 4:**
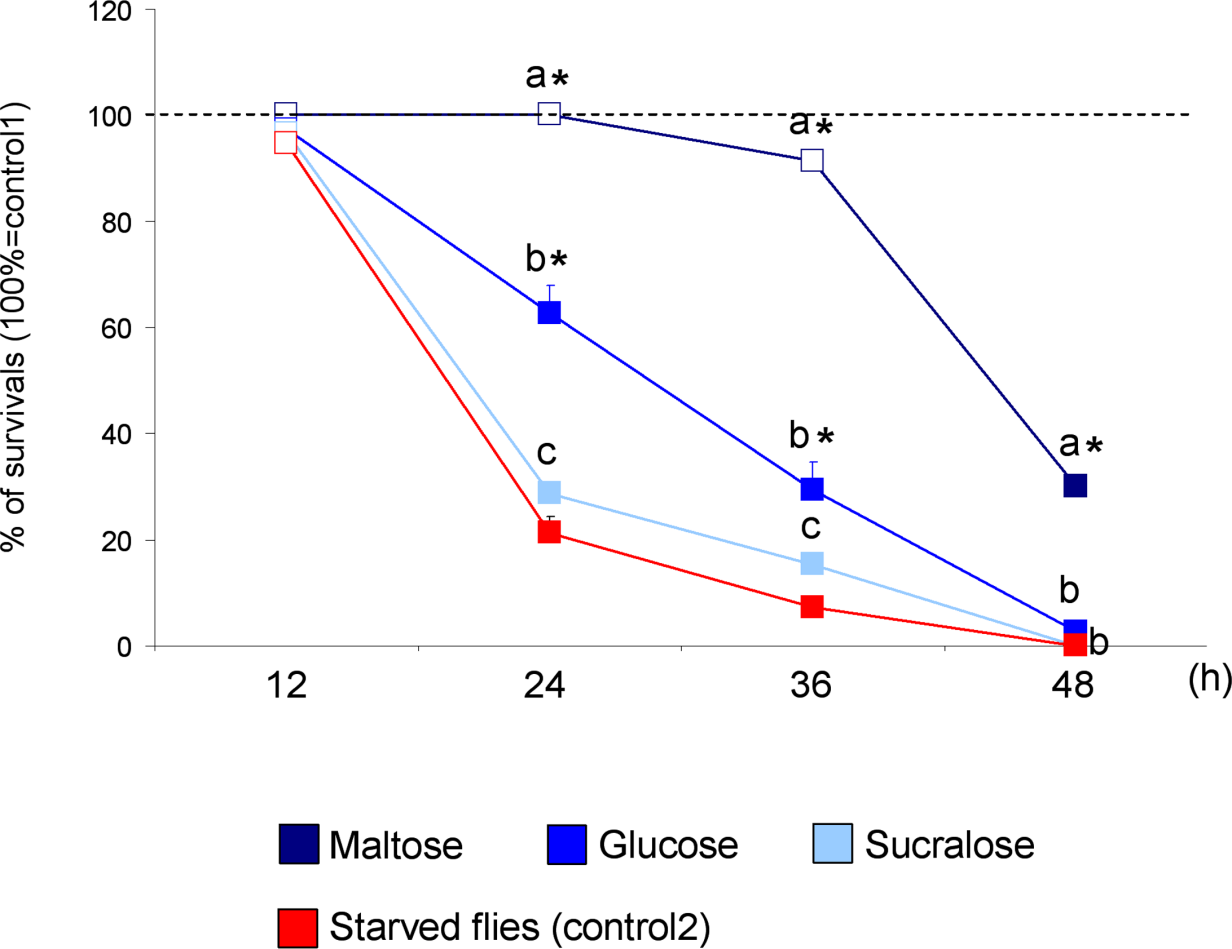
Survival results. Mean values ± s.e.m. of percentage of flies survived on each feeding substrate after 12, 24, 36 and 48 h fasting. Filled symbols indicate significantly different from control 1 (100% of insects at the end of feeding and start of starvation) (P<0.05; Duncan’s test). Different letters indicate significant differences between sugars at each time check (P<0.0001; Duncan’s test). (*) indicates significant differences from control 2 (starved flies) (P<0.01; Tukey test).

Mean values ± s.e.m. of the number of survived flies on each feeding substrate (standard diet with 43.82 mM of one of the test sugars) are shown in Fig 4. Two-way ANOVA revealed a significant interaction of Feeding substrate x Time on fly survival (F_[__8__,__40__]_ = 185.06; P<0.00001). In detail, post-hoc comparisons showed that the number of flies fed and survived on standard diet containing maltose was significantly lower compared to control 1 (end of feeding and start of starvation) only after a 48h fasting (P<0.01; Duncan’s test) and always significantly higher compared to the control 2 (starved flies) (P<0.0001; Duncan’s test) except for the 12h check; the number of flies fed and survived on a standard diet of glucose or sucralose was already significantly lower compared to the control after 24h of fasting (P<0.0001; Duncan’s test) compared to control 1. When fed on a glucose containing standard diet, the number of flies survived was significantly higher than control 2 (starved flies) at 24-36h (P<0.01; Duncan’s test), while no significant difference was found for sucralose at all time checks (P>0.05; Duncan's test). Besides, the number of flies fed and survived on maltose was significantly higher compared to the other two sugars and the survival success on sucralose was lower than on glucose, at 24-36h (P<0.05; Duncan’s test). These findings indicate that flies fed on a diet containing sucralose have a significantly shorter lifespan compared to glucose and maltose, while those fed on a diet containing maltose have a significantly longer lifespan, suggesting that the nutritional value of sugars tested is: maltose > glucose > sucralose.

### Effect of sugars on fatty acid composition

**Fig 5:**
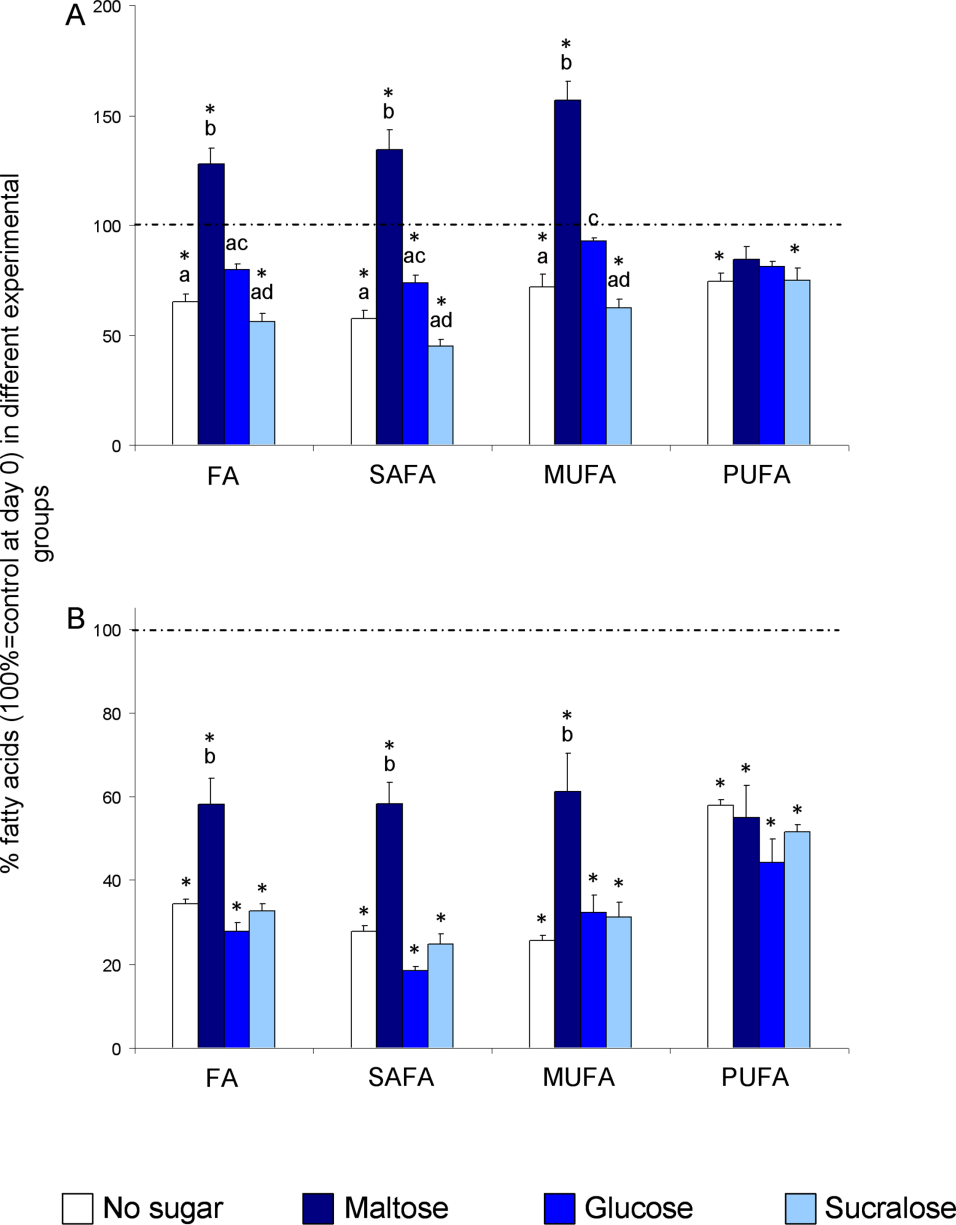
Metabolic results. Mean values ± s.e.m. of percentage of total FA, SAFA, MUFA and PUFA in experimental groups after feeding for 72h with different sugars (A) and fasting for 36h in alive flies (B). Asterisk indicates significant differences from the control (100% = day 0). Different letters indicate significant differences between sugars (P < 0.05; Tukey test or P < 0.05: Duncan’s test subsequent to one-way ANOVA).

One-way ANOVA revealed a significant effect of the feeding substrate on the amount of total FAs, SAFAs and MUFAs after 72h of feeding (F_[__4__,__25__]_>28.437; P<0.00001) than 36h of fasting (F_[__4__,__25__]_>8.5823; P<0.001) (Fig 5). Conversely, with PUFAs there is no significant variation (F_[__4__,__25__]_ = 1.474; P<0.05).

In particular, post-hoc comparisons showed that the amount of total FAs was higher in the flies fed 72h with maltose than those fed with glucose or sucralose, and also with respect to both controls, at day 0 and fed a diet without sugar (P<0.01; Tukey test). On the contrary, the amount of total FA was significantly lower when the sugar on the diet was sucralose (P<0.05; Tukey test), and not significantly different from those fed a diet without sugar (P<0.05; Tukey test). Finally, glucose-fed flies showed an intermediate FA level between those fed with maltose and sucralose (P<0.05; Tukey test). These findings indicate that the efficiency of lipid biosynthesis by sugars is the following: maltose > glucose > sucralose. When the sugar in the diet was sucrose, fructose or arabinose, as non-metabolizable sugar, we did not find any significant differences, if compared to maltose, glucose and sucralose, respectively. In fact, the amount of total FAs measured after 72h of feeding was 317.7±30.3 and 362.24±10.72 nmoles/fly for sucrose and maltose respectively (P>0.05; Tukey test), 243.2±15.7 and 226.9±6.82 nmoles/fly for fructose and glucose respectively (P>0.05; Tukey test), 189.8±8.2 and 158.94±10.28 nmoles/fly for arabinose and sucralose respectively (P>0.05; Tukey test). We focused our subsequent analyses on one sugar for each typology: maltose as disaccharide, glucose as monosaccharide and sucralose as a non-metabolizable sugar, in order to evaluate the variation in fatty acid profiles. Considering MUFAs (P<0.05; Duncan’s test) and SAFAs (P<0.05; Tukey test) the similar results were obtained, but PUFAs were not modified by any sugar (P<0.05; Tukey test), since their changes could be dependent only on the absorption from diets.

After 36h of fasting, the fly viability with maltose appeared to be in relation to lipid accumulation: in fact, all classes of fatty acids were consumed with different diets, in particular the ones without sugar or with sucralose, that produced the highest mortality rate.

Considering these lipid changes, we analysed the fatty acids profile with the different treatments, as described in detail in Table 1.

**Table 1:** Fatty acids profile in experimental groups after feeding for 72h with different sugars and fasting for 36h in alive flies. Different letters indicate significant differences in nmoles/fly of fatty acids between day 0 and values for the various sugars (P<0.05; Tukey test).

nmoles/fly		SAFA				MUFA			PUFA	
		12:0	14:0	16:0	18:0	14:1	16:1	18:1	n3 18:3	18:2
	Day 0	13.2±0.9a	39.1±2.9a	89.3±7a	9.9±0.8a	3.1±0.5a	19.9±1.5a	41.8±3.7a	13.2±0.9a	49.1±4.2a
Feeding 72h	No sugar	11.6±1.4a	20.1±1.9db	44.3±3.2b	5±0.2b	1.2±0.2b	15.8±1.3a	29.8±2.4b	13.1±0.5a	36.1±1.9b
	Maltose	25.6±5.5b	69.8±5.8c	91.2±7a	6.6±0.1c	3.8±0.3a	43.8±2.5b	54.1±3.1b	14±0.8a	41.6±2.7a
	Glucose	15±1.8ab	27.8±1.2b	55.3±3.7b	5.9±0.3bc	1.7±0b	21.8±1a	36.6±1.5a	13.4±0.9a	40.4±2.8a
	Sucralose	7±2.2a	11.4±1.2d	38.7±2.2b	5.3±0.3b	0.8±0.3b	13.1±0.6b	26.6±0.6b	12.6±0.3a	37.2±1.2b
Fasting 36h	No sugar	13.7±0.1a	0±0	19.6±0.1bd	4.5±0.2bc	0.2±0.1b	3.7±0.1b	12.7±1.3b	11.4±2.1a	27.5±1.2b
	Maltose	17.2±2.3c	23.3±0.8c	44.5±2.3c	4.2±0.1bc	1.1±0.2c	15±2.5c	23.6±3.3c	10.1±1.4a	26.5±3.7b
	Glucose	2.3±0.2b	3.3±0.1b	16.3±1.2b	3.6±0.1c	0.6±0.2b	5.8±0.5b	14.6±2b	8.6±1b	21±2.6b
	Sucralose	2.2±0.2b	2.5±0.6b	24.4±3.5d	5.2±1.3b	0.2±0b	3.1±0.1b	16.6±2.4b	10.5±0.1a	24±1.2b

A 72h feeding time with a diet containing maltose resulted in a significant increase about twice of medium chain acyl SAFA, such as 12:0 (lauric acid) and 14:0 (myristic acid) (P<0.05 and P<0.001, respectively; Tukey test). No difference of 16:0 (palmitic acid) and in a significant reduction of 30% of 18:0 (stearic acid) (P<0.001; Tukey test) respect to control day 0. Furthermore, MUFAs showed an significant increase for 16:1 (palmitoleic acid) (P<0.001; Tukey test) and 18:1 (oleic acid) (P<0.01; Tukey test), but not for 14:1 (myristoleic acid) (P<0.05; Tukey test), with respect to day 0 related to a higher desaturation rate.

## Discussion

Taste labellar sensilla in *Drosophila suzukii* were identified by comparison with those described in *D*. *melanogaster* on the basis of their length and distribution pattern (Fig 1)[20]. Given the aim of our study we chose to record the spike responses from the “l” type sensilla for two reasons: a) because of their length they are the most readily accessible to the “tip-recording” technique and, b) they contain 4 GRNs one of which, the S cell, is sensitive to sugars (mono-, di- and trisaccharides) [14,15]. In fact, the dose-response relationship that we found between the activity of "L" spike and the sugar concentration, suggests that "l" type sensilla in *D*. *suzukii* house a GRN sensitive to sugars.

It is known that feeding behaviour is mainly affected by two factors: food palatability and nutritional needs [24]; insects recognize and evaluate both the presence of an energy source (carbohydrates) and/or potentially harmful bitter compounds, that respectively produce an appetitive or aversive feeding behaviour [5,25,55,56].

In this study we have investigated for the first time in *D*. *suzukii* the differences of various dietary sugars in evoking taste responses from labellar sensilla both in terms of spike frequencies and levels of PER, that represent an effective measure of palatability [57]. We found that the disaccharide maltose evoked higher spike frequencies and induced higher PER responses with respect to the other treatment tested and, by activating the sweet-sensing GRN, may result the most palatable sugar among those tested. On the contrary, sucralose elicited very low spike frequencies and reflex responses, proving to be a weaker stimulus for the sugar-sensitive GRN and a less palatable sugar. Interestingly, glucose that was tested at an equimolar concentration of maltose and sucralose, elicited intermediate spike frequencies and levels of PER, suggesting that disaccharides possess lower sweetness than glucose in *D*. *suzukii* opposite to humans [58]. The relationship we found about taste sensitivity and palatability to different sugars is strengthened by the results on the amount of sugar eaten under double-choice conditions; in fact, maltose was the most consumed, while sucralose the least eaten, with glucose being intermediate. These results are consistent with previous studies on other insects. As a matter of fact, data from several laboratories on *D*. *melanogaster* show that: glucose and arabinose evoke both spike frequencies and levels of PER of similar strength [25]; the flies respond most robustly to disaccharides such as sucrose and maltose, while fructose and arabinose elicit intermediate levels of PER [48,57,59,60].

Taken together, our electrophysiological results as well as those on PER activity and amount of food intake, show that taste sensitivity and palatability for sugars are closely related, since those sugars which evoke a higher number of spikes also promote both a higher PER activity and food intake: this suggests that the more stimulant is a sugar the more appetitive it is for flies. A positive relationship between electrophysiological recordings and PER activity has already been described in *D*. *melanogaster* [25].

Still a matter of debate is whether taste sensitivity and palatability for sugars can be considered as indicators of their nutritional value [5,22–25,57]. Previous studies about *P*. *regina* suggest that there is no direct relationship between the taste of a compound and its nutritional value: sugars exist that are very stimulating, but have no nutritional value, and vice versa [61]. Most studies on *D*. *melanogaster* show that initially flies choose a sugar in strict accordance with taste or palatability; however, following deprivation of food, their preference shifts toward sugars with a higher nutritional content, sensed through postingestive mechanisms [23–25,57]. Instead, other studies suggest that taste sensitivity plays an important role in determining appropriate physiological and behavioural responses to nutritional value, particularly when flies live in environments where they encounter food sources with low nutritional contents [22]. In agreement with other reports, we found that flies fed on diets containing metabolizable sugars (maltose and glucose) live longer than those fed on diets with non-metabolizable sugars (sucralose) [57,59,62]. We found a positive relationship between the nutritional value of sugars and the responsiveness of sweet-sensitive GRN, with the disaccharide maltose being more capable to increase survival than the modified disaccharide sucralose, thus suggesting that maltose, as opposite to sucralose, is promptly hydrolysed to glucose which is the preferential substrate for DNL [63]. Accordingly, feeding glucose at equimolar concentration, hence providing half the amount of glucose with respect to maltose, showed lower survival rates. These data confirm previous studies on *D*. *melanogaster* where it was shown that survival capability is longer for flies fed equimolar amount with metabolizable disaccharides than for those fed with monosaccharides [64].

A possible explanation of the link between survival rate and palatability may rely on the very efficient capability to convert glucose to fat, rather than glycogen, by insects [33]. The advantages of storing lipids over carbohydrates as metabolic fuel include a higher caloric content per unit weight of substrate and thus the use of lipids as a primary metabolic substrate permits accumulation of a large reservoir of energy which may be used during periods of prolonged energy demand [33]. Besides, such high density energy stores certainly represent an advantage to the flight performance of winged insects [65].

In fact, data showed a significant increase of total FA levels by about 28% compared to control (day 0) with the diet containing maltose which indicates an efficient DNL; conversely, a decrease of 35%, 44% and 20% was shown with treatments without sugar, with sucralose and with glucose, respectively. Furthermore, a higher accumulation of 16:1, the most representative fatty acids widely used as biomarkers of DNL, was found in flies fed with maltose and glucose with respect to those fed with non-metabolizable sucralose. In the literature it is shown that insects possess a period of rapid lipogenesis during which dietary carbohydrates may be converted to lipid and stored in the fat body as triglycerides [29–31]. Moreover, the lipogenesis of insects is similar to that of mammalian tissues where the increase of plasma SAFA and 16:1 is not due to their higher food intake, but is the result of a DNL mainly in the liver [32,66]. Several studies have shown in humans that 16:1, formed by the activity of desaturation of 16:0, is correlated to the intake of carbohydrates [67]. For the first time, in this study we suggest that also in *D*. *suzukii* 16:1 is a reliable marker of DNL thereby influenced by glucose availability in the diet.

In conclusion, these results suggest that the ability to select different sugars on the basis of their palatability may favour the storage of a convenient energy reserves as fat derived from glucose via DNL, determining a longer survival capability during prolonged periods of fasting.

## Supporting information

S1 Fig Spike identification in response to 30 mM TCC. Figure provides data as they are displayed in different windows by the Clampfit 10.0 software: a) Samples of spike discharges following stimulation with 30 mM TCC. b) Spike identification by amplitude: 2 different spikes are shown: “M” and “S”, between cursors 3–4 and 1–2, respectively. c) Histogram showing spike amplitude classes. Vertical red dashed lines are the ideal boundaries of the spike types.
(PDF)

S2 Fig Spike identification in response to 100 mM maltose. Figure provides data as they are displayed in different windows by the Clampfit 10.0 software: a) Samples of spike discharges following stimulation with 100 mM maltose. b) Spike identification by amplitude: 3 different spikes are shown: “L”, “M” and “S”, between cursors 5–6, 1–2 and 3–4, respectively. c) Histogram showing spike amplitude classes. Vertical red dashed lines are the ideal boundaries of the spike types.
(PDF)

S3 Fig Spike frequency in l-type sensilla in response to sugars. Mean value ±s.e.m. of spike activity of l-type sensillum l_2_, l_4_ and l_5_ following stimulation with 100 mM maltose, glucose and sucralose. N = 6–7. One-way ANOVA revealed no effect of the sensillum number on the spike frequency (F_[__2__,__17__]_ <0.20499; p>0.05).
(PDF)

S4 Fig CAFE assay. a) Mean value±s.e.m. of amount of food intake in a no-choice condition. One-way ANOVA showed no effect of sugar on the amount of food intake (F_[__2__,__12__]_ = 0.13334; p = 0.9868). N = 5 vials/sug sugar. b) Sample of experimental arena for CAFE assay in a double-choice condition.
(PDF)
