# Supplementary figures and images for: Fat storage in Drosophila suzukii is influenced by different dietary sugars in relation to their palatability
Source: PLoS One. 2017 Aug 17;12(8):e0183173. doi: 10.1371/journal.pone.0183173 (PMC5560726; doi:10.1371/journal.pone.0183173)

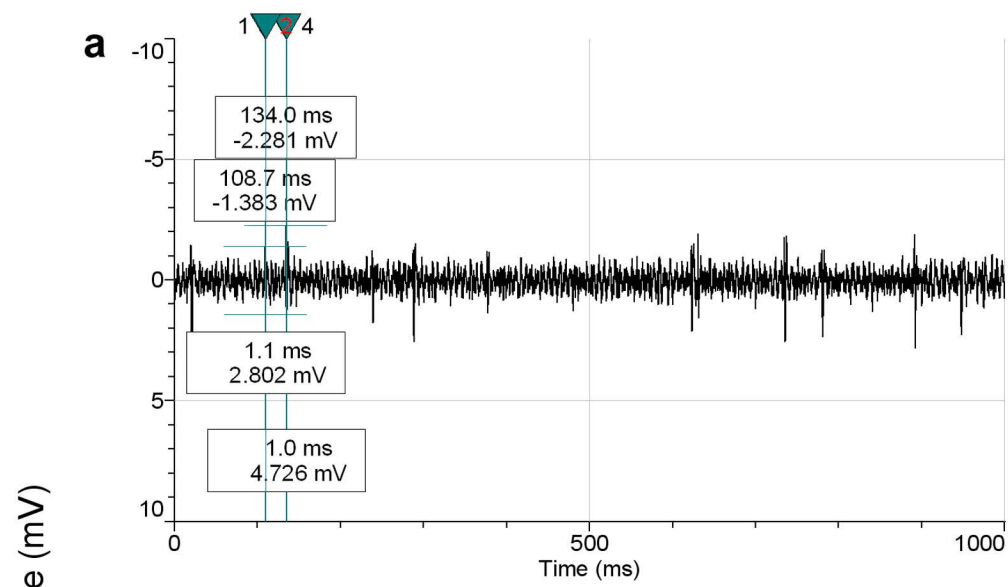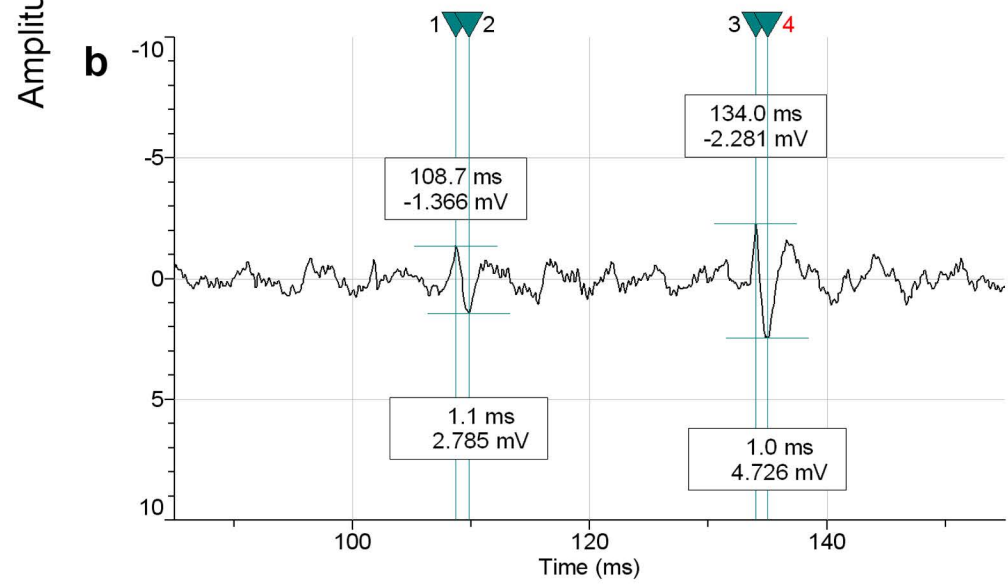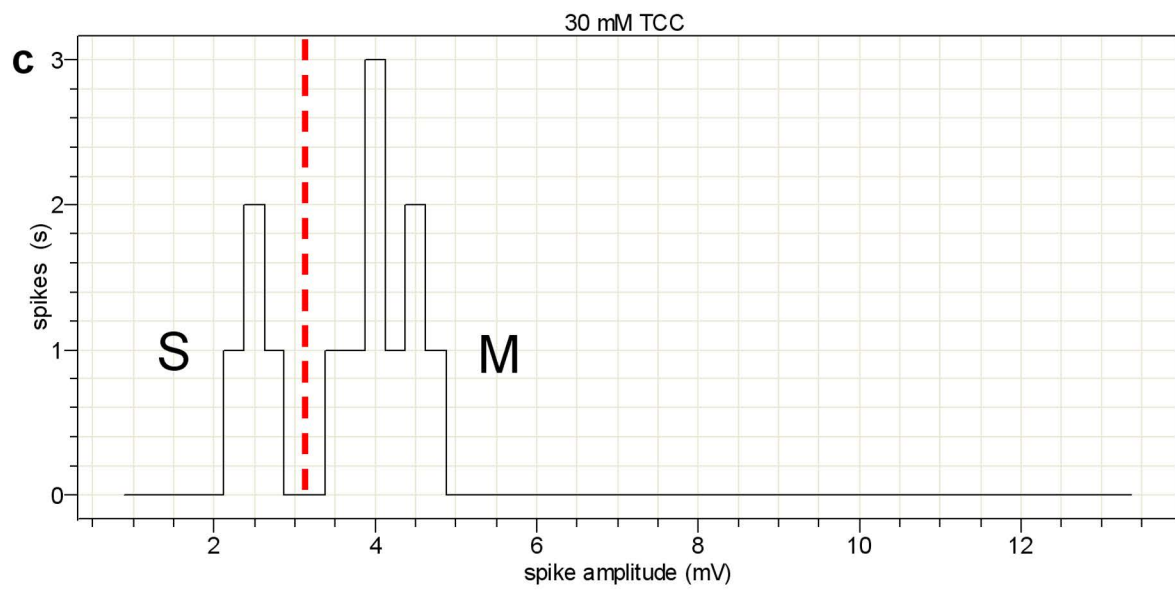

S1 Fig. Spike identification in response to 30 mM TCC.

Supplement: S1 Fig — Figure provides data as they are displayed in different windows by the Clampfit 10.0 software: a) Samples of spike discharges following stimulation with 30 mM TCC. b) Spike identification by amplitude: 2 different spikes are shown: “M” and “S”, between cursors 3–4 and 1–2, respectively. c) Histogram showing spike amplitude classes. Vertical red dashed lines are the ideal boundaries of the spike types. (PDF) [file pone.0183173.s001.pdf]

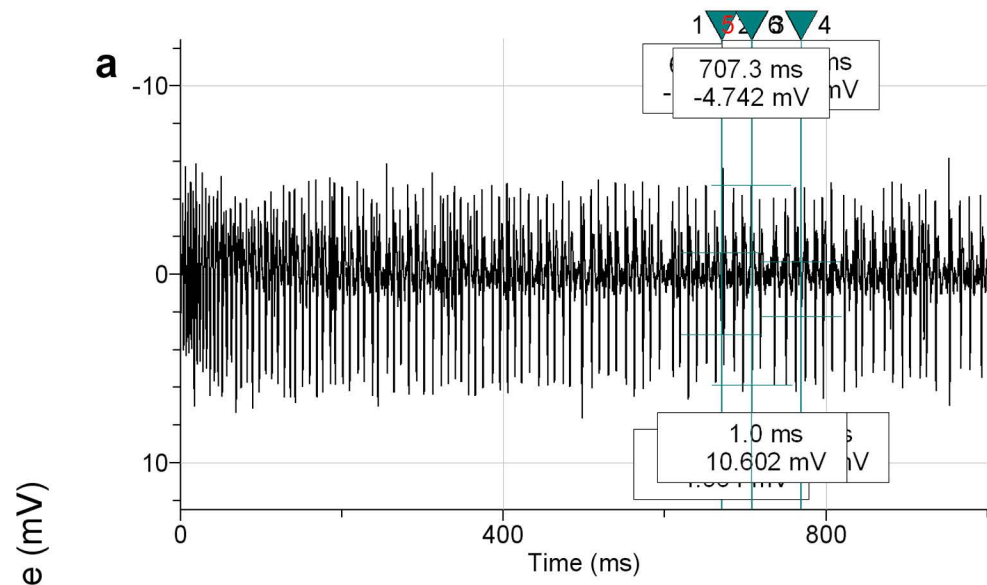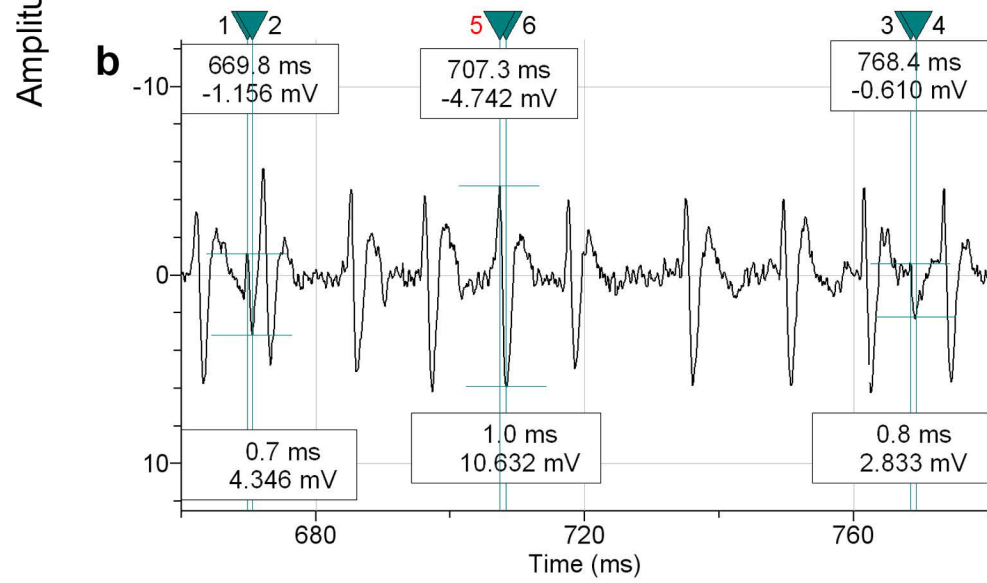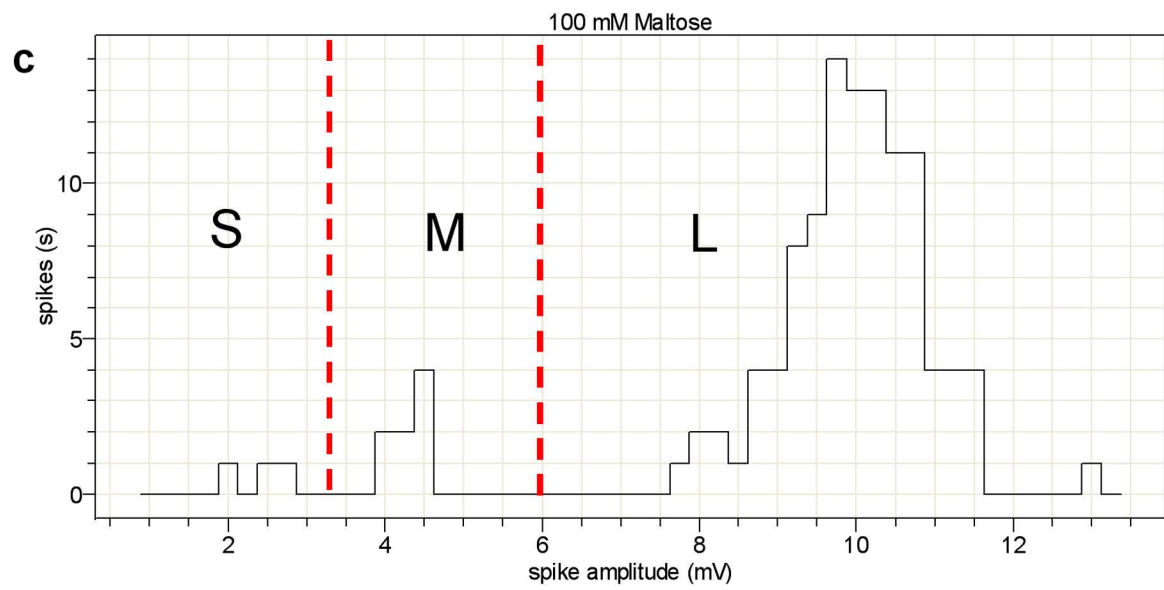

S2 Fig. Spike identification in response to 100 mM maltose.

Supplement: S2 Fig — Figure provides data as they are displayed in different windows by the Clampfit 10.0 software: a) Samples of spike discharges following stimulation with 100 mM maltose. b) Spike identification by amplitude: 3 different spikes are shown: “L”, “M” and “S”, between cursors 5–6, 1–2 and 3–4, respectively. c) Histogram showing spike amplitude classes. Vertical red dashed lines are the ideal boundaries of the spike types. (PDF) [file pone.0183173.s002.pdf]

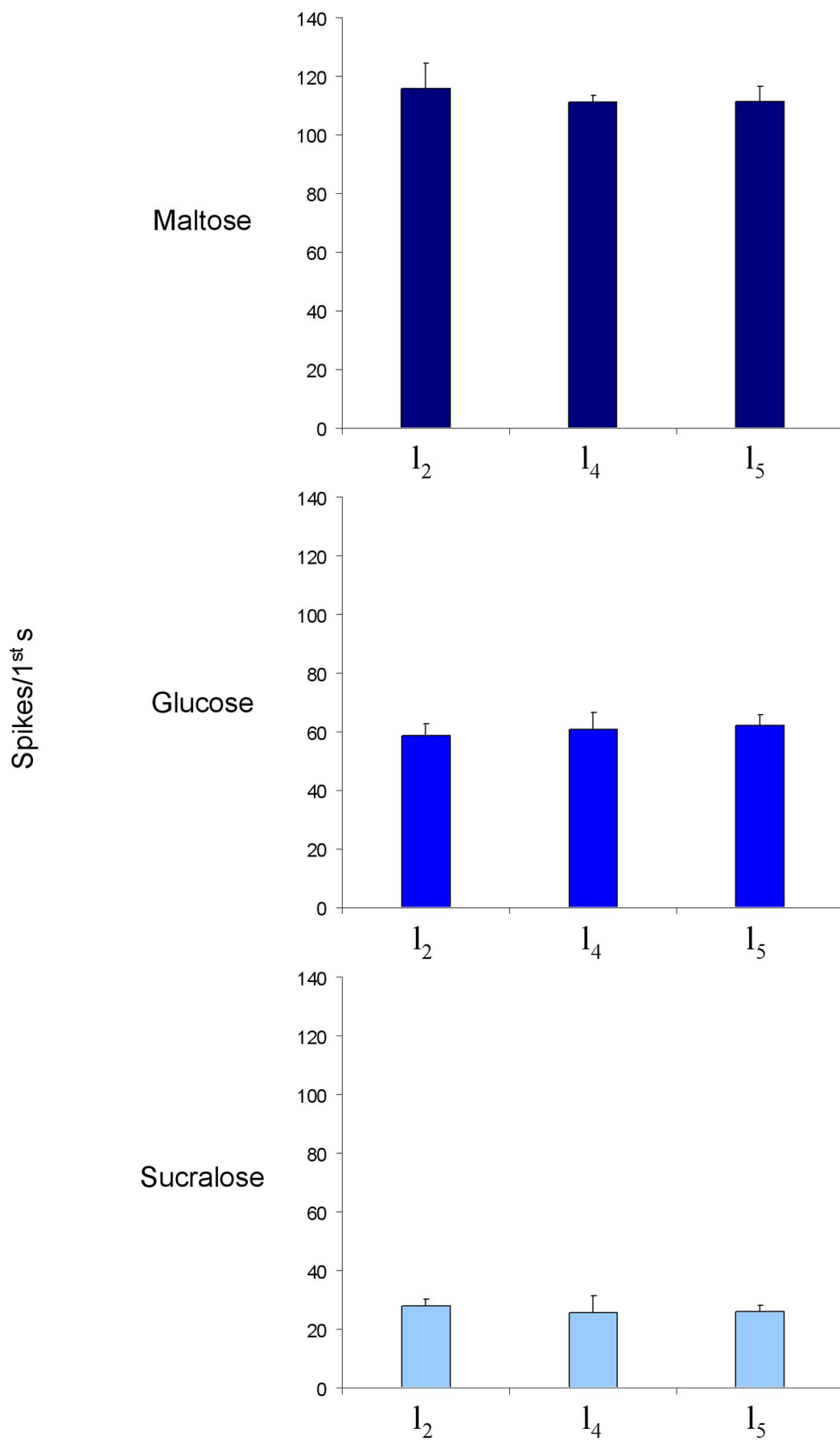

S3 Fig. Spike frequency in l-type sensilla in response to sugars.

Supplement: S3 Fig — Mean value ±s.e.m. of spike activity of l-type sensillum l2, l4 and l5 following stimulation with 100 mM maltose, glucose and sucralose. N = 6–7. One-way ANOVA revealed no effect of the sensillum number on the spike frequency (F[2,17] <0.20499; p>0.05). (PDF) [file pone.0183173.s003.pdf]

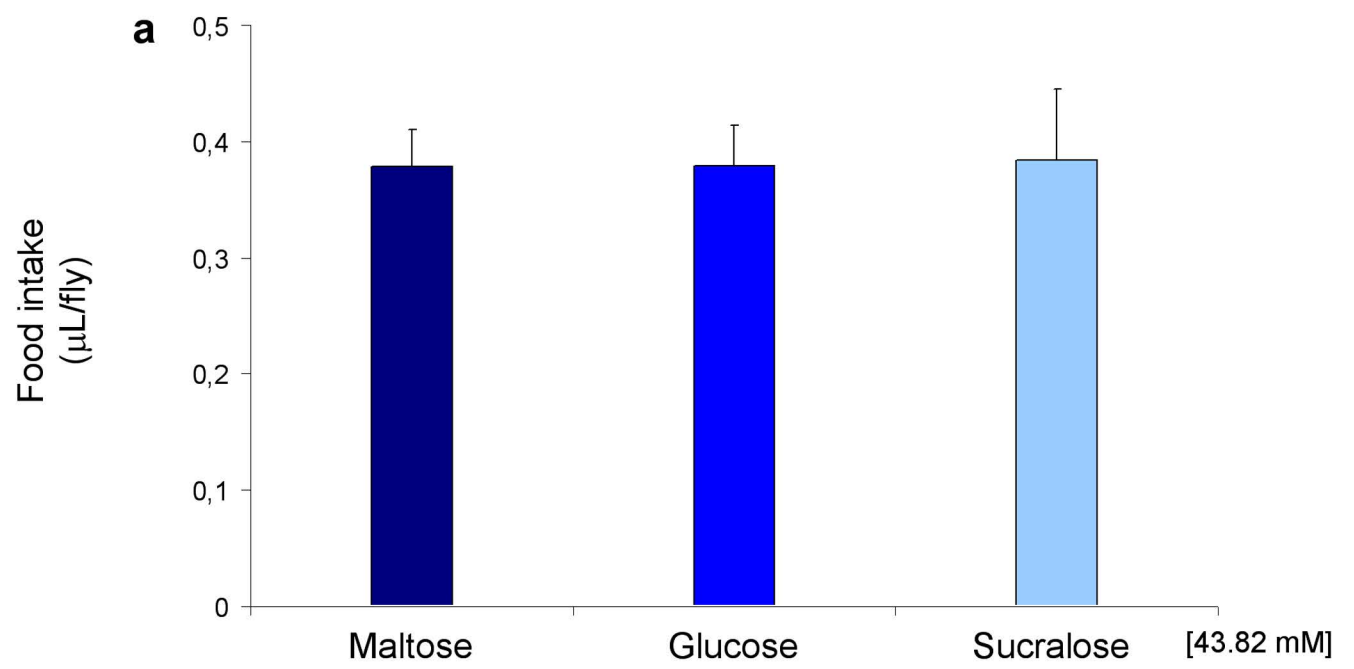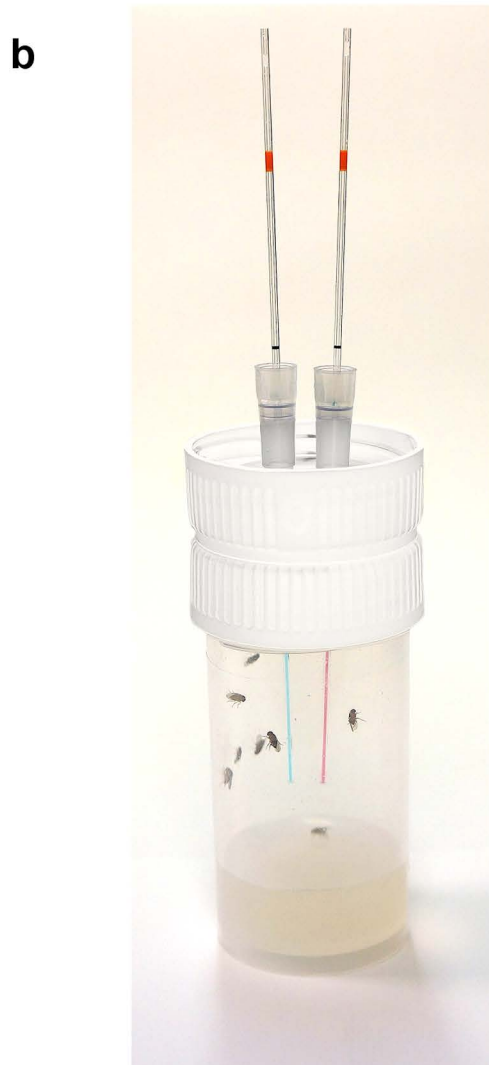

S4 Fig. CAFE assay.

Supplement: S4 Fig — a) Mean value±s.e.m. of amount of food intake in a no-choice condition. One-way ANOVA showed no effect of sugar on the amount of food intake (F[2,12] = 0.13334; p = 0.9868). N = 5 vials/sug sugar. b) Sample of experimental arena for CAFE assay in a double-choice condition. (PDF) [file pone.0183173.s004.pdf]
